# Tinnitus and dementia risk: a nationwide population-based case-control study

**DOI:** 10.1017/S0022215124001130

**Published:** 2024-12

**Authors:** Wesley W W Butt, Daniel R Wieland, Han Wang, Ching-Heng Lin, Jing-Jie Wang, Chien-Hsiang Weng

**Affiliations:** 1 Internal Medicine, University of Pennsylvania Health System, Philadelphia, PA, USA; 2 Department of Biomedical Engineering, University of Arizona, Tucson, AZ, USA; 3 Department of Neurology, Mayo Clinic College of Medicine & Science, Rochester, MN, USA; 4 Department of Neurology, Mayo Clinic Health System, Mankato, MN, USA; 5 Department of Medical Research, Taichung Veterans General Hospital, Taichung, Taiwan; 6 Department of Otolaryngology, Taichung Veterans General Hospital, Taichung, Taiwan; 7 School of Medicine, National Yang Ming Chiao Tung University, Taipei, Taiwan; 8 Department of Family Medicine, Brown University Warren Alpert Medical School, Providence, RI, USA; 9 Coastal Medical Lifespan, Providence, RI, USA

**Keywords:** tinnitus, dementia

## Abstract

**Objective:**

This study aimed to determine if a history of tinnitus is associated with the risk of developing dementia.

**Method:**

A nationwide population-based case–control study including all eligible adults in Taiwan.

**Results:**

A total of 15 686 patients were included in the study, with 7843 individuals making up each of the case and control groups. Patients with a history of tinnitus were associated with a statistically significant higher risk of being diagnosed with dementia before reaching 65 years old (50 years ≤ age <65 years) (adjusted odds ratio 2.68, 95 per cent confidence interval (CI) 1.19–6.05, *p* = 0.017). No statistical significance was found among those 65 years and older (adjusted odds ratio 1.17, 95 per cent CI 0.90–1.51, *p* = 0.235).

**Conclusion:**

A history of tinnitus was associated with a 168 per cent increased risk of being diagnosed with dementia in those aged 50–65 years old. This association was not significant in those older than 65 years.

## Introduction

Tinnitus, derived from the Latin word *tinnire* ‘to ring’, is a symptom characterised by the perception of sound in the absence of external stimuli. It is a common condition that affects millions of people, with a recent study estimating a contemporary prevalence of 1 in 10 adults in the USA.^1^ Despite its widespread prevalence, there is a lack of consensus on its exact underlying mechanisms. Human functional neuroimaging and other pathophysiological models have provided evidence that tinnitus-related activity changes in the brain involve both auditory and non-auditory structures, including the limbic system and the attention system, as well as other areas related to memory and emotion.^2,3^ Patients with chronic tinnitus often have a concomitant hearing impairment,^4^ and a growing body of evidence has linked tinnitus with cognitive impairment in adults.^5^

Dementia is a clinical syndrome characterised by progressive decline in two or more cognitive domains resulting in the loss of abilities to perform instrumental and/or basic activities of daily living.^6,7^ Alzheimer's dementia is the most common cause of dementia worldwide. Recent evidence has also indicated that sensory changes may precede the cognitive symptoms of Alzheimer's dementia, a progressive neurodegenerative disease, by several years.^8^ An association between hearing impairment and Alzheimer's dementia has previously been established, and a recently published nationwide population-based retrospective cohort study examining data from Taiwan in the early 2000s also suggested that tinnitus patients had a higher risk of developing Alzheimer's dementia.^9^ Previous studies have not examined whether the association between tinnitus and dementia may be age-dependent, and did not adjust for patients with coincident tinnitus and hearing loss. We sought to further evaluate this association using the latest available nationwide data from the National Health Insurance system.

## Materials and methods

**Figure 1.**
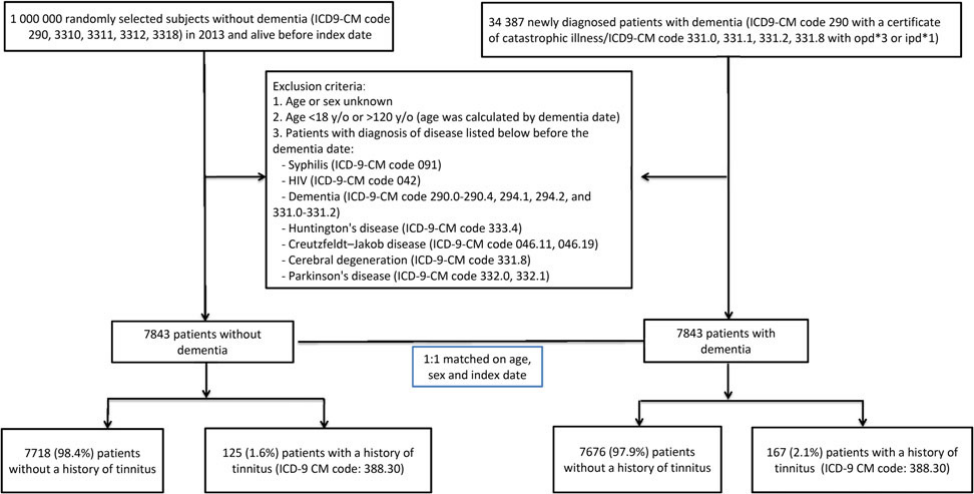
Flow diagram of participant selection and study design. ICD9-CM = International Classification of Diseases, Ninth Revision, Clinical Modification; opd = out-patient visit; ipd = in-patient hospitalisation; y/o = years old; HIV = human immunodeficiency virus.

We designed a large case–control study in which cases were defined as patients with a new diagnosis of dementia (diagnosed between 2006 and 2013) and no previous related medical history from the National Health Insurance Research Database (detailed exclusion criteria are described in the section on case selection and Figure 1). Controls without a dementia diagnosis were randomly selected and matched 1:1 to the cases based on age (by a margin of one month) and sex. We subsequently established the presence or absence of tinnitus prior to the diagnosis of dementia in the case group and the presence or absence of tinnitus using the same index date as the matched case patient in the control group.

### Taiwanese National Health Insurance Research Database

In 1995, the Taiwanese government established the National Health Insurance Program, providing coverage for the vast majority (99.6 per cent) of the country's population. The National Health Research Institute then created the National Health Insurance Research Database, a claims database also overseen by the Taiwanese Department of Health. Within the National Health Insurance Research Database there are multiple subset databases, including the Registry for Catastrophic Illness Patient Database and the Longitudinal Health Insurance Database. As dementia is administratively assigned to be a catastrophic illness, a diagnosed patient would therefore apply and be registered within the Registry for Catastrophic Illness Patient Database. The Longitudinal Health Insurance Database includes 1 million randomly selected persons designed to represent the total Taiwanese insured population, which numbered approximately 23 460 000 by the end of 2013.

### Case selection: dementia

Case patients were identified from the National Health Insurance Research Database as being newly diagnosed with dementia between 2006 and 2013. Diagnoses for dementia were registered using the Classification of Diseases, Ninth Revision, Clinical Modification code 290 with a certificate of catastrophic illness of 331.0, 331.1, 331.2, 331.8, which was crosslinked to the National Health Insurance Research Database. We used the presence of the code combined with a certificate of catastrophic illness to identify our initial pool of 34 387 potential patient cases.

We then excluded individuals whose ages and sexes were not known and those younger than 18 years or older than 120 years. Finally, we excluded those who presented a diagnosis of syphilis (International Classification of Diseases, Ninth Revision, Clinical Modification code 091), human immunodeficiency virus (International Classification of Diseases, Ninth Revision, Clinical Modification code 042), dementia (International Classification of Diseases, Ninth Revision, Clinical Modification codes 290.0–290.4, 294.1, 294.2 and 331.0–331.2), Huntington's disease (International Classification of Diseases, Ninth Revision, Clinical Modification code 333.4), Creutzfeldt–Jakob disease (International Classification of Diseases, Ninth Revision, Clinical Modification codes 046.11 and 046.19), cerebral degeneration (International Classification of Diseases, Ninth Revision, Clinical Modification code 331.8) or Parkinson's disease (International Classification of Diseases, Ninth Revision, Clinical Modification codes 332.0 and 332.1) before their diagnosis of dementia considering the possibility of having dementia-like symptoms or diagnosis. Based on these criteria, a total of 7843 cases was identified.

### Case–control match

A total of 7843 policy holders were selected as controls in a 1:1 match with the case group, randomly paired for age, sex and the same index date (the month and year of dementia diagnosis in the case group) from the 2013 version (consistent with our study time frame) of the Longitudinal Health Insurance Database. Similar to the case group, we excluded individuals whose ages and sexes were not known, those under 18 years or above 120 years old, those who were diagnosed with the diseases as defined in the case group and those who were deceased before the index date.

### Tinnitus

To identify patients with a tinnitus diagnosis, we searched for the International Classification of Diseases, Ninth Revision, Clinical Modification code 338.30. Additional criteria for inclusion were as follows: the same diagnosis in at least three out-patient visits or one in-patient diagnosis followed by either another in-patient or out-patient visit with the same diagnosis. Patients with a diagnosis strictly from a single in-patient visit were excluded. The first diagnosis of tinnitus must occur at least one year before the first dementia diagnosis in the case group or before the index date in the control group (Figure 1).

### Other adjustments

We adjusted for age, sex, a history of hypertension, diabetes, coronary artery disease, depression, hyperlipidaemia, alcohol dependence syndrome, thyroid disorders, hearing loss and radioactive iodine treatment. History of obesity was omitted from the analysis because of the small sample size.

### Statistics

We used the student's *t*-test to analyse continuous variables and the chi-square test to analyse categorical variables to observe differences in clinical characteristics between the case and control groups. A conditional logistic regression analysis was applied to examine the relationship between tinnitus and the risk of developing dementia, where we controlled for possible confounders. Statistical tests were all 2-sided using a significance level of 0.05 and reported using a 95 per cent confidence interval (CI) and/or *p* values. All analyses were run using SAS V.9.4.

This study was approved by the Institutional Review Board of Taichung Veterans General Hospital, Taichung, Taiwan (IRB #CE13152B-8).

## Results and analysis

A total of 15 686 patients were included in the study: 7843 in the case group and 7843 in the control group. The mean ages for those with dementia and those without dementia were 74.9 and 74.5 years, respectively. Between the case and control groups, there were significant differences (*p* < 0.05) in the proportion of patients who had histories of tinnitus, hearing loss, hypertension, diabetes, coronary artery disease, depression, alcohol dependence syndrome and thyroid disorders (Table 1).

**Table 1.** Clinical characteristics of study subjects with and without dementia

Variable*	Total (*n* = 15686)		Without dementia (*n* = 7843)		With dementia (*n* = 7843)		*p* value
	*N*	%	*n*	%	*n*	%	
Age, years (mean ± SD)	74.7 ± 11.3		74.5 ± 11.3		74.9 ± 11.3		─
Gender							─
– Female	8132	(51.8)	4066	(51.8)	4066	(51.8)	
– Male	7554	(48.2)	3777	(48.2)	3777	(48.2)	
History of tinnitus							0.013
– No	15394	(98.1)	7718	(98.4)	7676	(97.9)	
– Yes	292	(1.9)	125	(1.6)	167	(2.1)	
History of hearing loss							<0.001
– No	15408	(98.2)	7746	(98.8)	7662	(97.7)	
– Yes	278	(1.8)	97	(1.2)	181	(2.3)	
History of hypertension							<0.001
– No	8763	(55.9)	4632	(59.1)	4131	(52.7)	
– Yes	6923	(44.1)	3211	(40.9)	3712	(47.3)	
History of obesity							0.617
– No	15670	(99.9)	7834	(99.9)	7836	(99.9)	
– Yes	16	(0.1)	9	(0.1)	7	(0.1)	
History of diabetes							<0.001
– No	12147	(77.4)	6449	(82.2)	5698	(72.7)	
– Yes	3539	(22.6)	1394	(17.8)	2145	(27.3)	
History of coronary artery disease							<0.001
– No	13509	(86.1)	6842	(87.2)	6667	(85.0)	
– Yes	2177	(13.9)	1001	(12.8)	1176	(15.0)	
History of depression							<0.001
– No	15503	(98.8)	7819	(99.7)	7684	(98.0)	
– Yes	183	(1.2)	24	(0.3)	159	(2.0)	
History of hyperlipidaemia							0.422
– No	12791	(81.5)	6415	(81.8)	6376	(81.3)	
– Yes	2895	(18.5)	1428	(18.2)	1467	(18.7)	
History of alcohol dependence syndrome							<0.001
– No	15646	(99.7)	7841	(100.0)	7805	(99.5)	
– Yes	40	(0.3)	2	(0.0)	38	(0.5)	
Thyroid disorder							0.001
– No	15122	(96.4)	7604	(97.0)	7518	(95.9)	
– With hypothyroidism	102	(0.7)	34	(0.4)	68	(0.9)	
– With hyperthyroidism	133	(0.8)	57	(0.7)	76	(1.0)	
– With acquired hypothyroidism	5	(0.0)	3	(0.0)	2	(0.0)	
– Other	324	(2.1)	145	(1.8)	179	(2.3)	

^+^*t*-test; chi-square test for all other *p* values. Tinnitus: ICD 9CM 388.30; opd*3 or ipd*1; diagnosed 1 year prior to index date. Hearing loss: ICD 9CM 388.1, 388.2, 389.10, 389.0, 389.2, 389.9; opd*3 or ipd*1; diagnosed 1 year prior to index date. Hypertension: ICD 9CM 401; opd*3 or ipd*1; diagnosed 1 year prior to index date. Obesity: ICD 9CM 278.0; opd*3 or ipd*1; diagnosed 1 year prior to index date. Diabetes: ICD 9CM 250, A181; opd*3 or ipd*1; diagnosed 1 year prior to index date. Coronary artery disease: ICD 9CM 414.0, 414.8, 414.9; opd*3 or ipd*1; diagnosed 1 year prior to index date. Depression: ICD 9CM 311; opd*3 or ipd*1; diagnosed 1 year prior to index date. Hyperlipidaemia: ICD 9CM 272; opd*3 or ipd*1; diagnosed 1 year prior to index date. Alcohol dependence syndrome: ICD 9CM 303; opd*3 or ipd*1; diagnosed 1 year prior to index date. Hypothyroidism: ICD 9CM 243, 244.8, 244.9; opd*3 or ipd*1; diagnosed 1 year prior to index date. Hyperthyroidism: ICD 9CM 242; opd*3 or ipd*1; diagnosed 1 year prior to index date. Acquired hypothyroidism: ICD 9CM 242 and 244.0, 244.1, 244.2, 244.3; opd*3 or ipd*1; diagnosed 1 year prior to index date. SD = standard deviation; ICD 9CM = International Classification of Diseases, Ninth Revision, Clinical Modification; opd = out-patient visit; ipd = in-patient hospitalisation

After adjusting for age, sex, history of hypertension, diabetes, coronary artery disease, depression, hyperlipidaemia, alcohol dependence syndrome, thyroid disorders and hearing loss by logistic regression analysis (history of obesity was omitted from the analysis because of the small sample size[**Q3**]), having a history of tinnitus was associated with a statistically significant higher risk of being diagnosed with dementia before reaching the age of 65 years (50 years ≤ age <65 years) (adjusted odds ratio 2.68, 95 per cent CI 1.19–6.05, *p* = 0.017). No statistical significance was found among those 65 years and older (adjusted odds ratio 1.17, 95 per cent CI 0.90–1.51, *p* = 0.235) (Table 2).

**Table 2.** Adjusted odds ratio of dementia associated with tinnitus

Variable	Dementia		
	Adjusted odds ratio	95% CI	*p* value
Age 50–65 years (***n*** = 1806)			
– No history of tinnitus	1.00	–	–
– History of tinnitus	2.68	1.19–6.05	0.017
Age over 65 years (***n*** = 13 324)			
– No history of tinnitus	1.00	–	–
– History of tinnitus	1.17	0.90–1.51	0.235

Odds ratio was adjusted for sex, age, history of tinnitus, hypertension, diabetes, coronary artery disease, depression, hyperlipidaemia, alcohol dependence syndrome, thyroid disorders, hearing loss and radioactive iodine treatment by logistic regression analysis. History of obesity was omitted from the analysis due to the small sample number. CI = confidence interval

## Discussion

In this case–control study of 15 868 patients, we found an increased risk of dementia associated with a history of tinnitus in patients aged 50–65 years (adjusted odds ratio 2.68, *p* = 0.017). Consistent with current literature, dementia diagnoses were also associated with a number of conditions and illnesses, including hearing loss (*p* < 0.001), hypertension (*p* < 0.001), diabetes (*p* < 0.001), coronary artery disease (*p* < 0.001), depression (*p* <0.001), alcohol dependence syndrome (*p* < 0.001) and thyroid disorders (*p* < 0.001).

A previous study by Cheng *et al*. established a 1.675-fold increase in the risk of early-onset dementia among those aged 30–64 years in the same population using the National Health Insurance Research Database.^10^ However, to our knowledge, no study has expounded whether this association extends between tinnitus and dementia in adults aged 65 years or older, especially in an East-Asian population. Our study reveals that tinnitus is not associated with dementia in older patients (*p* = 0.235) and supports the assertion that this correlation exclusively operates in the context of early-onset dementia.

The pathophysiological relationship between the development of dementia and tinnitus remains insufficiently explored. Numerous mechanisms and external factors potentially underlie our results. For example, although tinnitus does not cause hearing loss, loss of hearing can amplify the severity of tinnitus.^11^ Hearing loss has long been established as a significant risk factor with incident all-cause dementia, with Lin *et al*. proposing that the former can contribute to the depletion of cognitive reserve.^12–14^ Echoing this idea, a systematic review found that subjective tinnitus is associated with significantly diminished function in cognitive performance for general short-term memory, response time, processing accuracy, and general learning and retrieval tasks.^15^

Cognitive reserve has been postulated as one of the keys to understanding the disconnect between obvious brain pathologies and remarkably unimpaired neurophysical performance in patients.^16^ Consequently, tinnitus may exhaust this cognitive reserve. In one out of five patients, tinnitus can be so severe that it contributes to depression, insomnia, anxiety, irritability and hyperacusis, which can all serve as risk factors, lending greater susceptibility to patients to develop cognitive impairment in the future.^17–20^

As previous work has suggested, there is a significant likelihood that both tinnitus and dementia are clinical indicators of shared, underlying dysfunction in the patient's neurochemistry. The root causes of tinnitus have yet to be illuminated. Researchers have proposed that the ear's ringing, whooshing or buzzing perception stems from three primary sources: alterations to the brain's temporal patterns, an uptick in abnormal spontaneous firing rates in the auditory pathway and restructuring of the tonotopic maps.^21–23^ Imaging techniques such as positron emission tomography, diffusion magnetic resonance imaging (MRI) and BOLD-fMRI in human and animal studies have mapped a significant portion of the central mechanisms involved in tinnitus.^3^

Both auditory and non-auditory networks have been found to play a role in failing to compensate for symptoms via maladaptive homeostatic plasticity. These areas include the inferior colliculus, dorsal cochlear nucleus, paraflocculus lobe in the cerebellum, posteroventral cochlear nucleus, medial prefrontal cortex, basal ganglia, dorsal prefrontal regions, parietal cortex, medial and caudolateral orbital cortex, insula, posterior thalamus, anterior and posterior cingulate cortex, amygdala, parahippocampus, hippocampus and nucleus accumbens.^23–37^ Given that the amygdala, hippocampus and posterior cortices are the three most affected sites in MRI studies of early-onset Alzheimer's, there are indeed confounding multifaceted pathways that may connect dementia to tinnitus.^38^ For example, in a small percentage of the population, traumatic brain injuries or forms of brain damage can directly trigger both tinnitus and dementia.^39–41^ Whether tinnitus precisely precedes or develops concurrently concerning cognitive impairment remains vital to study.

Although Alzheimer's disease is still the most common cause of dementia in young-onset dementia, other aetiologies, such as frontotemporal dementia and vascular dementia, are more prevalent in this group.^42^ Even in Alzheimer's disease patients, atypical phenotypes, such as posterior cortical atrophy, are more common in the young-onset patient population. Our study showed a history of tinnitus is associated with increased risk of dementia only in the younger (<65 years) population, but not in the older population. This could suggest the effect of tinnitus varies in different age groups. Alternatively, this could be related to the underlying differences in the pathophysiology and anatomical areas affected by the aetiologies of dementia.

### Strength and limitations

As the National Health Insurance Research Database encompasses nearly the entirety of the Taiwanese population, one of the greatest strengths of this study is the large and representative sample size. The nature of insurance claims discourages selection bias, recall bias and under-reporting. Furthermore, we accounted for confounders in our case–control study by implementing logistic regression analysis for age, sex, hearing loss, hypertension, coronary artery disease, depression, hyperlipidaemia, alcohol dependence syndrome and thyroid disorders (history of obesity was omitted because of the small sample size).

- Tinnitus, a condition impacting 10 per cent of US adults, coincides with the growing prevalence of dementia in the aging population, but there remains a scarcity of evidence exploring the connection between these two conditions across various age groups
- The Taiwanese National Health Insurance Research Database was used to conduct a population-based, retrospective case–control study in all eligible adults
- Dementia patients were 1:1 matched to control patients with the same age, sex and index date (month and year of dementia diagnosis in the case group) with further adjustment for known risk factors
- Between the case and control groups there were significant differences (*p* < 0.05) in the proportion of patients who had histories of tinnitus, hearing loss, hypertension, diabetes, coronary artery disease, depression, alcohol dependence syndrome and thyroid disorders
- A history of tinnitus was associated with a 168 per cent increased risk of being diagnosed with dementia in patients aged 50–65 years, but the association was not significant in those older than 65 years

The source of strength of this study also coincides with a limitation, as Taiwan's ethnic population is effectively homogenous, consisting of overwhelmingly Han Chinese. Additionally, without imaging or laboratory data (e.g. brain MRI scans, genetic results, cerebrospinal fluid Aβ42 or tau protein), diagnoses of dementia do not elucidate the severity of the disease or its developmental timing in individuals in the database. Likewise, the severity, progressive onset and specificity of tinnitus are not fully captured by the International Classification of Diseases, Ninth Edition code. For instance, unlike subjective tinnitus, objective tinnitus is not technically a true hearing disorder because the hearing organs and neuropathology are not strictly dysfunctional. Although very few tinnitus patients have this mechanical problem, our inability to exclude them may slightly skew results. Furthermore, by the nature of observational studies, we can only determine the association between, not the biological causality of, tinnitus and dementia.

## Conclusions

In this East-Asian nationwide case–control study, a history of tinnitus was associated with a 168 per cent increased risk of being diagnosed with dementia in those aged 50–65 years old. This association was not significant in those older than 65 years (*p* = 0.235). The hope is that future physicians and patients will use these findings when studying the predictive factors of early dementia. For now, the connection between tinnitus and dementia has been primarily explored through correlative studies. Future pathophysiological and prospective longitudinal studies may be beneficial to uncover causality and any underlying biochemical mechanisms between the two.
